# Systematic review and meta-analysis of age-related differences in instructed emotion regulation success

**DOI:** 10.7717/peerj.6051

**Published:** 2018-12-18

**Authors:** Brooke Brady, Ian I. Kneebone, Nida Denson, Phoebe E. Bailey

**Affiliations:** 1 School of Social Sciences and Psychology, Western Sydney University, Sydney, NSW, Australia; 2 Department of Clinical Psychology, University of Technology Sydney, Sydney, NSW, Australia

**Keywords:** Older adults, Emotion regulation, Response modulation, Cognitive change, Meta-analysis

## Abstract

The process model of emotion regulation (ER) is based on stages in the emotion generative process at which regulation may occur. This meta-analysis examines age-related differences in the subjective, behavioral, and physiological outcomes of instructed ER strategies that may be initiated *after* an emotional event has occurred; attentional deployment, cognitive change, and response modulation. Within-process strategy, stimulus type, and valence were also tested as potential moderators of the effect of age on ER. A systematic search of the literature identified 156 relevant comparisons from 11 studies. Few age-related differences were found. In our analysis of the subjective outcome of response modulation strategies, young adults used expressive enhancement successfully (*g* = 0.48), but not expressive suppression (*g* = 0.04). Response modulation strategies had a small positive effect among older adults, and enhancement vs suppression did not moderate this success (*g* = 0.31 and *g* = 0.10, respectively). Young adults effectively used response modulation to regulate subjective emotion in response to pictures (*g* = 0.41) but not films (*g* = 0.01). Older adults were able to regulate in response to both pictures (*g* = 0.26) and films (*g* = 0.11). Interestingly, both age groups effectively used detached reappraisal, but not positive reappraisal to regulate emotional behavior. We conclude that, in line with well-established theories of socioemotional aging, there is a lack of evidence for age differences in the effects of instructed ER strategies, with some moderators suggesting more consistent effectiveness for older compared to younger adults.

## Introduction

Early theories of aging assumed that growing older was associated with global declines in physical, cognitive, and emotional health (Banham, 1951; Buhler, 1968; Cumming & Henry, 1961). However, recent research suggests that physical and cognitive declines in older age are offset by improved emotional health (Charles & Carstensen, 2007). Older age is associated with decreased negative affect (Carstensen et al., 2011; Grühn, Kotter-Grühn & Röcke, 2010; Stone et al., 2010), lower rates of clinical anxiety and depression (see review by Piazza & Charles (2006)), and equal if not greater positive affect (Mroczek & Kolarz, 1998). Emotional wellbeing may be considered the silver lining of older age, and age-related differences in emotion regulation (ER), that is the selection of which emotions are experienced, when they are experienced, and how they are expressed over time (Gross, 1998; Gross, Sheppes & Urry, 2011), might be one factor underpinning these gains. Indeed, successful regulation of emotion is critical for adaptive functioning across the lifespan and has been linked to improved mental health (Gross & Muñoz, 1995), physical health (Sapolsky, 2007), and relationship satisfaction (Murray, 2005). ER can be spontaneous or instructed. Instructed ER involves the provision of a simple written or verbal instruction that is designed to guide the way one processes and/or responds to emotional events. The present systematic review and meta-analysis is the first to examine age-related differences in the success of instructed ER strategies.

The most widely cited model of ER is Gross’ (1998) process model which posits five distinct processes (each encompassing unique ER strategies) organized according to their temporal position in the emotion-generative process. First, *situation selection* involves selecting one situation in lieu of other options in order to give rise to the most desirable emotional outcome. Next, *situation modification* involves altering aspects of the present situation in order to change the emotional impact. Following this, *attentional deployment* processes require motivated shifts in attention toward or away from aspects of the emotional experience in order to influence the emotional response. Once an aspect of the emotional event is attended to, *cognitive change* strategies are employed in order to select one of many possible interpretations of the event with the goal of altering the emotional impact. Once meaning has been assigned, *response modulation* processes directly control the experiential, behavioral or physiological response to the event. No aging research to date has assessed age differences in situation selection or situation modification capacities using *instructed* ER procedures. As such, only studies assessing the efficacy of attentional deployment, cognitive change, and response modulation strategies are included in the current meta-analysis.

Three meta-analyses have examined the effectiveness of instructed ER more generally (Aldao, Nolen-Hoeksema & Schweizer, 2010; Augustine & Hemenover, 2009; Webb, Miles & Sheeran, 2012). Of these, only Webb, Miles & Sheeran (2012) examined age-related differences in strategy success. Self-report, behavioral, and physiological outcomes of experimentally instructed attentional deployment, cognitive change, and response modulation were examined across 190 studies. It was shown that age did not moderate ER success. However, participants in most of the studies were aged between 18 and 30 years of age and few recruited older adults. Since then, the body of literature investigating age-related differences in ER success has grown significantly. This makes a meta-analysis of age effects both feasible and critical to gaining a true understanding of potential age-related differences in ER.

The influential socioemotional selectivity theory (SST; Carstensen, 1992; Carstensen, Isaacowitz & Charles, 1999) suggests that as older adults realize that time remaining in life is limited, they begin to prioritize emotionally meaningful goals and actively pursue more positive emotional encounters (although see Almeling et al., 2016). This is in direct contrast to earlier stages of the adult lifespan when time is seen as expansive and one is motivated to prioritize the acquisition of knowledge in order to achieve material goals. This theory suggests greater motivation in older age to maintain emotional wellbeing and engage in successful emotional regulation. In particular, there is considerable evidence for an age-related positivity effect involving increased effort to focus attention and memory away from negative information and toward positive information (see review by Reed, Chan & Mikels (2014)).

Like SST, the model of strength and vulnerability integration (SAVI; Charles, 2010) recognizes the importance of time left to live in motivating ER. However, SAVI also incorporates time lived and emotional experience as important factors influencing one’s ER processes. SAVI suggests that greater emotional knowledge and experience navigating the stressors of daily life may lead to advantages for many older adults in terms of their ability to successfully regulate emotion. SAVI also attempts to explain some of the documented age-related deficits in emotion processing. Specifically, it suggests that reduced physiological flexibility may result in an age-related increase in the debilitating consequences of highly arousing and inescapable emotional stimuli.

## Age-Related Differences in Emotion Regulation

### Attentional deployment

While a number of strategies have been identified under the umbrella of attentional deployment, the two studies to date that have assessed instructed attentional deployment have investigated the impact of distraction strategies only, and results suggest that this type of regulation may remain intact, or may even become more effective in older age. Compared to young adults, older adults are better able to decrease negative affect when asked to focus their attention away from an upsetting film clip to instead focus on a positive autobiographical memory (Phillips et al., 2008). Similarly, older (and not younger) adults report reduced negative mood when asked to focus on the emotionally neutral content of a sad film (Lohani & Isaacowitz, 2014). Increased skin conductance levels (SCLs) in response to a sad film indicate that both young and older adults find it equally difficult or effortful to direct attention away from negative content (Lohani & Isaacowitz, 2014). Interestingly, recent evidence suggests that young adults are less likely to prefer the use of attentional deployment strategies compared to both middle-aged and older adults, despite holding stronger beliefs in the effectiveness of distraction strategies (Livingstone, Castro & Isaacowitz, 2018).

### Cognitive change

Studies assessing age differences in instructed cognitive change have typically explored three within-process strategies: detached reappraisal, positive reappraisal, and negative reappraisal. *Detached reappraisal* requires participants to view the content of an emotive stimulus with objectivity and/or detached interest. *Positive reappraisal* requires participants to focus on the positive aspects of emotional stimuli and imagine possible positive outcomes that may come from the emotional event. *Negative reappraisal* involves focusing on the negative aspects of an emotional stimulus and thinking about possible negative consequences of the depicted event.

Compared to younger adults, older adults may be more successful at using positive reappraisal strategies to regulate emotion, and are more likely to prefer positive reappraisal strategies (Livingstone, Castro & Isaacowitz, 2018). They also experience less negative affect when using positive reappraisal while watching sad films (Lohani & Isaacowitz, 2014; Shiota & Levenson, 2009). A systematic review has also revealed that older adults use positive reappraisal strategies at a commensurate or lesser rate than younger adults, but may consistently benefit more from this strategy (Nowlan, Wuthrich & Rapee, 2015). In contrast, older adults are less successful than young adults at using detached reappraisal to decrease unpleasant emotions after viewing negative images (Opitz et al., 2012; Pedder et al., 2016; Tucker et al., 2012), and sad and disgusting films (Shiota & Levenson, 2009). While one study has found that older adults are significantly better than younger adults at increasing negative affect in response to negative pictures using negative reappraisal (Opitz et al., 2012), other studies have found no age-related differences in the effect of negative reappraisal when responding to sad pictures (Opitz et al., 2014) or sad films (A. H. Coats, 2007, unpublished data).

Physiological outcomes of cognitive change strategies suggest that positive reappraisal may be more adaptive than detached reappraisal as we age. When asked to positively reappraise sad films, older adults experience reduced SCLs, while young adults experience increased SCL (Lohani & Isaacowitz, 2014), suggesting more adaptive responding among the former. Similarly, an average of eight physiological indices (cardiac interbeat interval, SCL, finger pulse amplitude, pulse transmission time to the finger, pulse transmission time to the ear, finger temperature, respiration depth, and mean arterial blood pressure) showed that older adults, but not younger adults, experience reduced physiological reactivity when positively reappraising sad and disgusting films (Shiota & Levenson, 2009). In contrast, *detached reappraisal* is effective in reducing physiological reactivity to upsetting films among young adults, but not older adults (Shiota & Levenson, 2009). A behavioral outcome of the same regulation strategy was reduced reactivity of the *zygomaticus* (smiling) and *corrugator* (frowning) muscle regions, as measured by facial electromyography (EMG) when viewing positive and negative images (Pedder et al., 2016). However, it is noted that the older adults had lower facial reactivity at baseline, and thus had less emotional expression to down-regulate relative to their younger counterparts.

### Response modulation

Studies that have investigated instructed response modulation processes have typically done so in two ways. First, *expressive suppression* requires participants to limit their emotional expression such that someone watching would not know that they were experiencing any emotion. Second, *expressive enhancement* involves expressing as much emotion as possible such that someone watching would clearly know how they were feeling.

There is little evidence for age-related differences in the subjective emotional outcome of response modulation processes. Studies report no age differences in negative mood following instructions to *suppress* the expression of emotion in response to film-induced sadness (Lohani & Isaacowitz, 2014; Shiota & Levenson, 2009), injustice (Phillips et al., 2008), and disgust (Kunzmann, Kupperbusch & Levenson, 2005). Similarly, Pedder et al. (2016) found no age differences in subjective happiness/unhappiness when participants were asked to minimize facial expressions of emotion in response to both positive and negative films. There also does not appear to be any age-related difference in subjective reactivity when asked to *enhance* emotional expression in response to positive and negative music (Vieillard, Harm & Bigand, 2015) and negative images (C. M. Van Reekum & D. Lloyd, 2016, unpublished data). However, relative to young adults, older adults self-report experiencing more intense negative affect when asked to *suppress* emotion expression in response to high arousal negative music (Vieillard, Harm & Bigand, 2015).

All studies assessing behavioral outcomes using facial EMG have found that both young and older adults are equally able to reduce their facial reactivity when instructed to suppress their expression of emotion. This has been demonstrated in response to positive and negative music (Vieillard, Harm & Bigand, 2015), sad films (Lohani & Isaacowitz, 2014), and negative images (Pedder et al., 2016). However, physiological data shows that suppression of negative emotion in response to music results in higher systolic blood pressure among older, but not younger adults (Vieillard, Harm & Bigand, 2015). They suggest this may indicate that the suppression of negative emotional content is more cognitively and physiologically demanding for older adults than for younger adults. The same study found no age-related difference in facial EMG across age groups when asked to enhance emotion by exaggerating facial expression.

The effect of expression suppression on age differences in SCLs is unclear. Young and older adults experience increased SCLs when asked to suppress their expressivity in response to a sad film (Lohani & Isaacowitz, 2014). Other studies have found decreased SCLs among both age groups when asked to suppress negative emotion in response to negative music (Vieillard, Harm & Bigand, 2015) and disgusting films (Kunzmann, Kupperbusch & Levenson, 2005) with the former showing some evidence that older adults experience a longer lasting decrease in SCL.

## Moderators of Emotion Regulation Effectiveness

### Within-process strategy type

Webb, Miles & Sheeran (2012) identified important within-process differences in strategy effectiveness for young adults. Similarly, SAVI suggests that older adults may regulate emotion more effectively when using strategies that cater to age-related strengths (such as the positivity bias) and capitalize on emotional experience. It may be that older adults’ tendency to attend to and appraise positive aspects of their environment means that positively reappraising emotive content requires less effort relative to other cognitive change strategies. Older adults’ increased life experience may also provide more opportunity to practice using positive reappraisal strategies effectively, as well as providing a greater number of perceived positive outcomes to draw upon. Further, dynamic integration theory (DIT; Labouvie-Vief, 2008) suggests that older adults may be more likely to use ER strategies that reduce demands on cognitive resources, such as detached reappraisal. The present meta-analysis will determine whether there are differences in the effectiveness of within-process strategies, including within cognitive change processes.

### Stimuli type

Older adults may perform better on lab-based tests when the stimuli are more naturalistic (Phillips et al., 2014). Thus, age-related differences in ER may be minimized with film as opposed to pictorial stimuli. This is because stimuli in real life are usually multimodal rather than being presented in a single modality. Alternatively, older adults find it harder to regulate more intense emotions (Vieillard, Harm & Bigand, 2015), suggesting that they may find it more difficult to regulate emotions in response to film relative to pictorial stimuli. This is consistent with DIT (Labouvie-Vief, 2008), which suggests that intense emotional content may result in the breakdown of systems that regulate the integration of cognition and emotion. The current meta-analysis will help to disentangle whether older adults experience more difficulty regulating emotions induced by multimodal film relative to unimodal pictorial stimuli.

### Valence

Dynamic integration theory (Labouvie-Vief, 2008) also suggests that age-related reductions in cognitive resources and reduced psychophysiological flexibility with age make it more difficult for older adults to represent emotional situations in complex ways. Older adults are less likely than young adults to dwell on complex emotions (more often negative than positive) that require effortful consideration of meaning and relevance. It is not clear if this tendency to avoid negative emotions that require complex processing will translate into greater difficulty regulating negative emotions. However, the positivity effect suggests that older adults may be adept at both up-regulating positive affect and down-regulating negative affect (Isaacowitz, 2006). The present meta-analysis will determine whether older adults differ in their ability to regulate positive vs negative stimuli.

### The current study

Theoretical and empirical evidence to date suggests that there are both age-related similarities and differences in ER processes. The present systematic review and meta-analysis aimed to elucidate any age-related differences in a number of ways. First, differences between young and older adults in the effectiveness of different ER processes (attentional deployment, cognitive change, response modulation) were determined. Second, in light of previous findings that effects of different emotional outcomes do not necessarily mirror aggregated process effect sizes (Webb, Miles & Sheeran, 2012), we also aimed to quantify potential age-related differences in the *subjective*, *behavioral*, and *physiological* outcomes of each ER process. Third, we tested whether within-process strategy, stimulus type, or valence moderated differences in ER success for young vs older adults.

## Method

### Eligibility criteria

Published data and unpublished theses were eligible for inclusion in the meta-analysis if they were in English, and met four inclusion criteria: (1) studies were required to include at least one age comparison between an older adult group with a mean age of 59 years or higher, and a young adult group with a mean age of 40 years or younger. Studies with only one age group were excluded from the analysis (Gyurak et al., 2012; Urry et al., 2009). Where more than two age groups were included in a study (Shiota & Levenson, 2009), only the youngest and oldest groups contributed; (2) because the focus is on healthy, neuro-typical community-dwelling older adults, studies that investigated ER among samples with clinical psychopathology, neurodegenerative disorder, or adults from assisted living facilities were excluded; (3) studies had to manipulate (rather than measure) ER, using a form of attentional deployment, cognitive change, or response modulation as outlined in Gross’ (1998) process model of ER. Studies also had to include a control condition (“no regulation” or “just watch”) to allow for calculation of strategy effectiveness compared to a baseline regulation state. Within-process strategies that had been measured in only one study to date (i.e., focused breathing; D. Pedder, 2016, unpublished data) could not be included; and (4) studies had to include a measure of emotion needed following instructed ER. Acceptable measures included self-reports of emotional state (e.g., ratings of discrete emotions, ratings of general positive/negative affect), measures of expressive behavior (e.g., coding of facial expressivity, facial EMG) and physiological measures (e.g., cardiovascular measures, skin conductance). Outcome measures that could not be easily interpreted in terms of ER success (e.g., blood oxygen level dependent changes in brain regions, gaze aversion) were excluded.

### Information sources

Studies were identified via three sources: (1) a computerized search of social scientific databases (Web of Science, PsycINFO, Scopus) for articles published before November 1, 2016; (2) screening reference lists of relevant articles; and (3) a call for unpublished data adhering to eligibility criteria via the APA Division 20 listserv.

### Search

Electronic searches were completed using the search terms “older adults,” elderly, seniors, geriatrics, ageing, aging, old, older, “life span,” “late life” AND reapprais*, suppress*, distract*, ruminat*, mindful*, acceptance AND “mood regulation,” “emotion regulation,” “affect regulation” (in the title, abstract or keywords). Duplicates and articles not available in English were removed, and articles were subject to the four eligibility criteria based on sample characteristics and experimental practices.

### Study selection and data collection process

The literature search was conducted by Brooke Brady. The identified articles and theses were screened for duplicates and articles not available in English were removed. Data were then subjected to the four eligibility criteria outlined above. Brooke Brady and Phoebe E. Bailey independently coded the instructed ER strategies in each eligible study according to the ER strategy categories outlined in Gross’s (1998) process model of ER. Webb, Miles & Sheeran’s (2012) taxonomy of ER strategies was also used to help identify within-process strategies. An interrater reliability analysis using Cohen’s Kappa statistic was performed to determine consistency among raters. This analysis indicated near perfect agreement between raters: Kappa = 0.97 (*p* < 0.001), 95% CI [0.91–1.03]. A single coding disagreement was resolved by discussion. Where experiments included manipulations of more than one emotional regulation strategy and/or more than one control condition, all possible experimental and control comparisons were included in the present analysis. Brooke Brady also coded each study for the following characteristics: (a) sample size and age of young adult and older adult groups; (b) to-be-regulated emotion valence; (c) emotive stimulus type (pictures, film); and (d) outcome measure(s).

### Summary measures and synthesis of results

Meta-analysis explored age differences in attentional deployment, cognitive change and response modulation. All analyses were carried out using Comprehensive Meta-Analysis Version 3.3.07 (Borenstein et al., 2014). Specifically, subgroup analyses investigated differences in age group effects (Borenstein et al., 2009). Moderators of effects within age groups were also analyzed. We deemed this approach appropriate as it allows for a richer understanding of age differences as well as the relative ER strengths and weaknesses of young and older adults. Because there is variation in the measurement scales used to assess common continuous emotional outcomes across studies (e.g., all included studies assess subjective emotional reactivity, however, they use different psychometric scales), the standardized mean difference (*g*) is used in the present meta-analysis as the effect size summary statistic (Higgins & Green, 2009). The standardized mean difference expresses the size of the effect in each study relative to the variability observed in that study (Higgins & Green, 2009). This ensures that the results of studies are standardized to the same scale before they are statistically combined. Where multiple comparisons from a single experiment led to the same participants being included more than once in the calculation of an effect size, we divided the *N* by the number of comparisons from the single study contributing to the comparison to reduce the impact on the overall effect size. We used a random-effects model to allow for population parameters to vary across studies, and to allow the findings to generalize beyond the specific studies included in the meta-analysis (Borenstein et al., 2014; Hedges, 1992; Hedges & Vevea, 1998). Hedge’s *g* (0.2 = small, 0.5 = medium, and 0.8 = large) is similar to Cohen’s *d* but adjusts for small sample size bias. Effect size direction was determined according to the success of strategies in line with individual strategy aims. For example, expression suppression aims to reduce the experience of emotion by limiting one’s behavioral expression of emotion. Therefore, effect direction for this strategy was deemed positive if emotional reactivity decreased following expressive suppression instructions, and was deemed negative if emotional reactivity increased following expression suppression instruction. We examined the heterogeneity in effect sizes using the *I*^2^ statistic (i.e., the proportion of total variability in an effect size that is due to true heterogeneity rather than sampling error; Higgins & Thompson, 2002). To control for any bias or imprecision of *I*^2^ in small meta-analyses we also report confidence intervals as recommended by Von Hippel (2015). The *Q*-test based on analysis of variance was used to determine statistically significant differences between mean weight effect sizes (i.e., the between-subgroups portion of the variance), as well as variance in effect sizes within age groups.

## Results

### Study selection and characteristics

**Figure 1:**
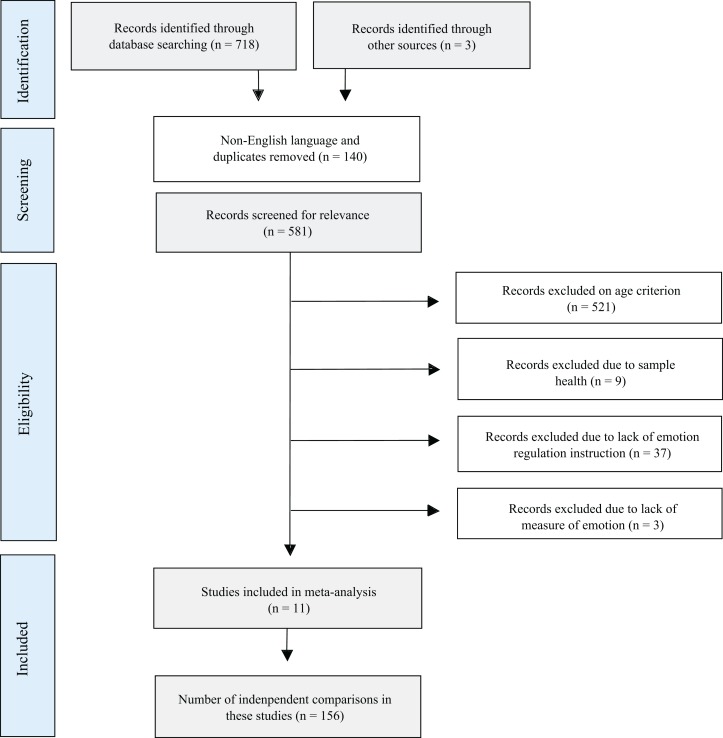
Flow diagram depicting search strategy.

Figure 1 shows the search strategy for the review presented according to the PRISMA reporting guidelines (Liberati et al., 2009). Of the 721 articles, theses, and other unpublished data sets initially identified, 11 met criteria for inclusion in the meta-analysis. Table 1 presents study characteristics of the included articles.

**Table 1:** Characteristics of effect sizes to be included in the meta-analysis.

Study	Valence	Stimulus type	Strategy type	Young adults				Older adults			
				*N* (*M* age)	Effect size (*g*)			*N* (*M* age)	Effect size (*g*)		
					Subjective	Behavioral	Physiological		Subjective	Behavioral	Physiological
Opitz et al. (2012)	Negative	Pictures	Negative reappraisal	16 (19.25)	0.29			15 (59.87)	0.68		
			Positive reappraisal	16 (19.25)	−0.03			15 (59.87)	0.08		
Opitz et al. (2014)	Negative	Pictures	Negative reappraisal	30 (19.5)	0.83	0.45	0.92^†^	30 (61.9)	0.76	0.86	0.52^†^
			Detached reappraisal	30 (19.5)	0.51	1.07	−0.33^†^	30 (61.9)	0.33	1.18	−0.06^†^
Pedder et al. (2016)	Positive	Pictures	Expression suppression	35 (23.2)	0.92	1.14		35 (71.63)	0.51	1.13	
			Detached reappraisal	35 (23.2)	1.32	1.23		35 (71.63)	0.89	1.18	
	Negative	Pictures	Expression suppression	35 (23.2)	0.18	0.76		35 (71.63)	0.25	0.70	
			Detached reappraisal	35 (23.2)	0.49	0.70		35 (71.63)	0.35	0.70	
Vieillard, Harm & Bigand (2015)	Positive	Music	Expression enhancement	31 (31.0)	0.07	1.13	0.09^†^	30 (64.0)	0.20	0.45	0.12^†^
			Expression suppression	31 (31.0)	0.25	0.00	0.41^†^	30 (64.0)	−0.04	0.37	0.40^†^
	Negative	Music	Expression enhancement	31 (31.0)	0.64	0.77	0.12^†^	30 (64.0)	0.13	0.64	−0.06^†^
			Expression suppression	31 (31.0)	−0.49	0.12	0.28^†^	30 (64.0)	−0.12	0.00	0.45^†^
Shiota & Levenson (2009)	Negative	Film	Detached reappraisal	22 (25.5)	0.56	0.34		24 (64.8)	−0.02	0.21	
			Positive reappraisal	26 (25.3)	0.04	0.28		24 (64.5)	0.31	0.02	
			Expression suppression	48 (25.4)	0.42	0.56		48 (64.65)	0.09	0.41	
Phillips et al. (2008)	Negative	Film	Expression suppression	16 (22.7)	0.19	0.64		16 (72.2)	0.07	1.04	
			Attentional deployment	16 (22.7)	0.30	0.00		15 (72.2)	1.11	1.03	
Lohani & Isaacowitz (2014)	Negative	Film	Attentional deployment	42 (18.5)	0.12	0.45	−0.45	48 (71.42)	0.47	0.19	−0.22
			Positive reappraisal	42 (18.5)	0.18	0.29	−0.52	48 (71.42)	0.70	0.16	−0.08
			Expression suppression	42 (18.5)	0.21	0.56	−0.49	48 (71.42)	0.42	0.34	−0.12
A. H. Coats, unpublished thesis	Negative	Film	Positive reappraisal	104^▿^	0.07			42^▿^	−0.09		
			Negative reappraisal	55^▿^	0.20			94^▿^	−0.12		
D. Pedder, unpublished thesis—Chapter 5	Positive	Film	Expression suppression	40 (22.0)	0.15	0.65	−0.04	40 (71.3)	0.22	0.62	0.09
	Negative	Film	Expression suppression	40 (22.0)	−0.21	0.99	−0.22	40 (71.3)	0.00	0.70	0.14
	Positive	Film	Expression suppression	40 (22.0)	−0.23	0.50	−0.13	40 (71.3)	0.16	0.54	0.14
	Negative	Film	Expression suppression	40 (22.0)	−0.01	0.82	−0.11	40 (71.3)	−0.15	0.58	0.00
	Positive	Film	Expression suppression	40 (22.0)	−0.16	0.25	−0.10	40 (71.3)	0.12	0.27	0.15
D. Pedder, unpublished thesis—Chapter 6	Negative	Film	Expression suppression	40 (22.0)	−0.22	0.58	−0.22	40 (71.3)	0.00	0.50	0.14
			Detached reappraisal	40 (22.0)	−0.01	0.97	−0.20	40 (71.3)	0.00	0.43	−0.12
C. M. Van Reekum & D. Lloyd, unpublished thesis	Negative	Pictures	Expression suppression	20 (27.0)	−0.03			67 (69.4)	−0.14		
			Expression enhancement	20 (27.0)	0.58			67 (69.4)	0.49		

**Notes:**

† Indicates a mean effect size calculated for two separate physiological outcome measures. Positive effect sizes indicate the emotion regulation strategy was successful in line with strategy aims. Negative effect sizes indicate the emotion regulation strategy was not effective in line with strategy aims.

▿ Indicates that age range only was provided for this study; 18–30 years for young adults, 60–80 years for older adults.

### Overall effect sizes

Table 2 displays the overall effect of each ER process, as well as the effect within each outcome type, averaged across age group. Cognitive change processes had a small to medium positive overall effect, while response modulation had a small positive effect, and attentional deployment had no reliable effect. For subjective outcomes, response modulation processes had a small positive effect and both cognitive change and attentional deployment had a small to medium positive effect. For behavioral outcomes, attentional deployment processes had a small to medium positive effect and both cognitive change and response modulation had medium positive effects. ER processes had no reliable effect on physiological outcomes.

**Table 2:** Effects of emotion regulation processes, averaged across age group.

Process	Outcome	*N*e	*g*	95% CI	*I*^2^	*Z*	*p*
Attentional deployment	All	8	0.25	[−0.01–0.51]		1.89	0.06
	Subjective	4	0.43	[0.10–0.77]	64.89	2.54	0.01
	Behavioral	4	0.37	[0.04–0.70]	64.55	2.21	0.03
	Physiological	NA	NA	NA	NA	NA	NA
Cognitive change	All	52	0.36	[0.24–0.48]		5.90	<0.001
	Subjective	24	0.33	[0.19–0.47]	73.01	4.59	<0.001
	Behavioral	16	0.61	[0.42–0.81]	76.27	6.25	<0.001
	Physiological	12	0.03	[−0.22–0.28]	76.61	0.24	0.81
Response modulation	All	94	0.26	[0.18–0.33]		7.06	<0.001
	Subjective	34	0.12	[0.03–0.22]	64.03	2.61	0.01
	Behavioral	30	0.57	[0.47–0.67]	60.40	10.94	<0.001
	Physiological	30	0.05	[−0.04–0.14]	37.21	1.09	0.28

**Note:**

*N*e, number of effect sizes included in calculation; *g*, effect size; NA, insufficient data to allow for calculation.

### Subgroup analysis

Table 3 displays the subjective, behavioral, and physiological effects of each ER process for young and older adults, as well as the age group comparison statistics. No evidence for age-related differences was found.

**Table 3:** Age-related effects on subjective, behavioral, and physiological outcomes of emotion regulation processes.

Process	Outcome	*N*e	Effect size (*g*)		*Q*	*p*
			Young	Older		
Attentional deployment	Subjective	4	0.19	0.67	2.62	0.11
	Behavioral	4	0.24	0.54	0.43	0.51
	Physiological	NA	NA	NA	NA	NA
Cognitive change	Subjective	24	0.35	0.31	0.09	0.76
	Behavioral	16	0.65	0.57	0.16	0.69
	Physiological	12	−0.02	0.09	0.15	0.70
Response Modulation	Subjective	34	0.11	0.14	0.07	0.79
	Behavioral	30	0.62	0.53	0.71	0.40
	Physiological	30	−0.04	0.12	3.55	0.06

**Note:**

*N*e, number of effect sizes included in calculation; NA, insufficient data to allow for calculation.

However, our analysis revealed significant variation in effect sizes within the young and older adult subgroups on subjective and behavioral outcomes of cognitive change and response modulation strategies (see Table 4). This warranted exploration of moderator variables to attempt to account for variance within subgroups not otherwise explained.

**Table 4:** Tests of effect size variation from study to study within young and older adult subgroups.

Process	Outcome	Young adults			Older adults		
		*Q*	d*f*	*p*	*Q*	d*f*	*p*
Attentional deployment	Subjective	0.36	1	0.55	3.38	1	0.07
Cognitive change	Subjective	41.64	11	<0.001	43.43	11	<0.001
	Behavioral	25.58	7	<0.001	36.33	7	<0.001
	Physiological	32.55	5	<0.001	12.34	5	0.03
Response modulation	Subjective	62.06	16	<0.001	29.07	16	0.02
	Behavioral	43.45	14	<0.001	27.96	14	0.01
	Physiological	22.70	14	0.07	17.29	14	0.24

### Moderator analysis

#### Within-process strategy type

Within-process strategy type was explored as a moderator of ER success within each age group.

**Figure 2:**
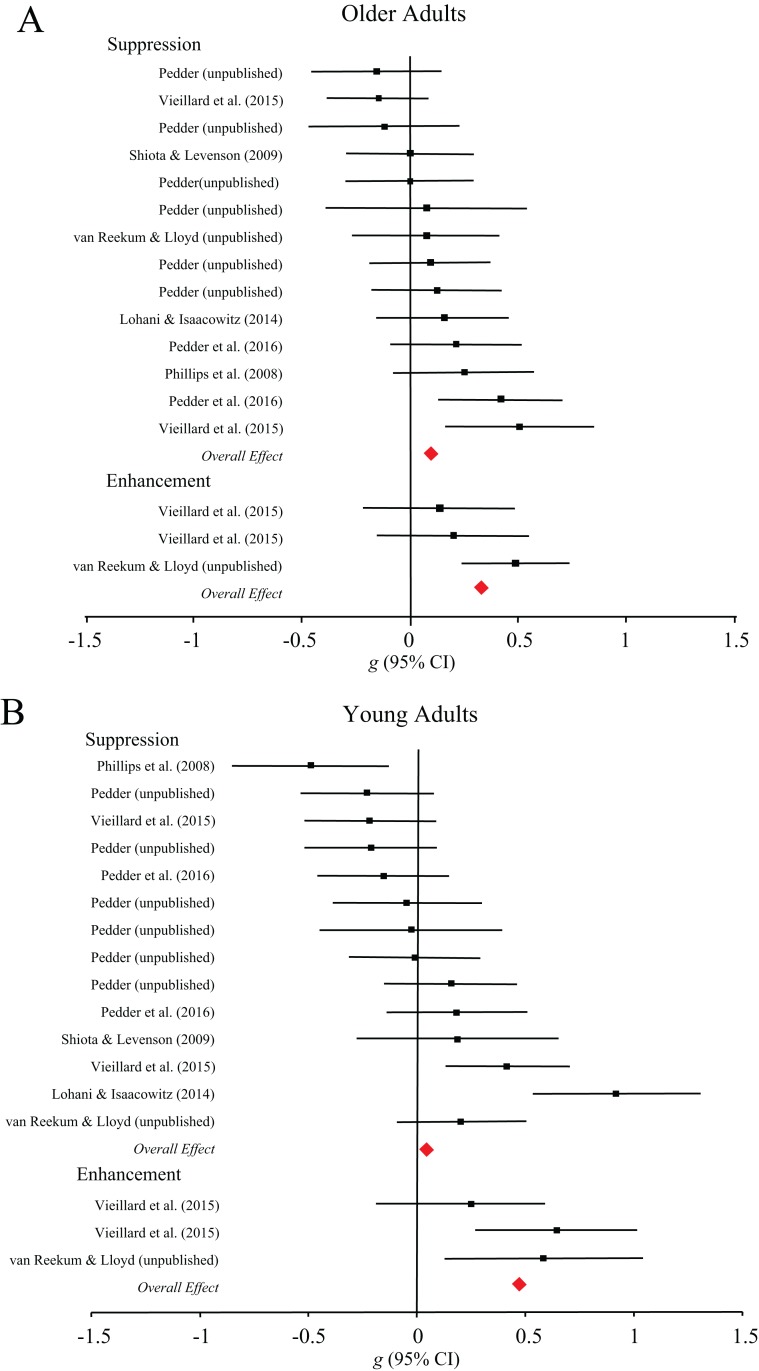
Forest plots demonstrating effect sizes for the subjective outcomes of expressive suppression and expressive enhancement strategies for older (A) and younger (B) adults.

**Subjective outcomes.** There were insufficient comparisons to include perspective taking (*k* = 1) in the moderator analysis for cognitive change strategies. There were no differences in the effect of detached reappraisal, negative reappraisal or positive reappraisal among young adults, *Q*(2) = 3.77, *p* = 0.15, or older adults, *Q*(2) = 0.21, *p* = 0.90. For response modulation processes (see Fig. 2), expression enhancement was effective among young adults (*g* = 0.48, 95% CI [0.11–0.86]), whereas expression suppression was not (*g* = 0.04, 95% CI [−0.12–0.21]), *Q*(1) = 4.46, *p* = 0.04. We found no evidence for differences in enhancement (*g* = 0.31, 95% CI [0.08–0.53]) or suppression (*g* = 0.10, 95% CI [0.01–0.21]) strategy success among older adults, *Q*(1) = 2.59, *p* = 0.11.

**Behavioral outcomes.** There were insufficient comparisons to include negative reappraisal (*k* = 1) or perspective taking (*k* = 1) in the moderator analysis for cognitive change strategies. For young adults, detached reappraisal had a large positive effect on behavioral outcomes (*g* = 0.80, 95% CI [0.51–1.10]), but positive reappraisal had no reliable effect (*g* = 0.28, 95% CI [−0.12–0.68]), *Q*(1) = 4.20, *p* = 0.04. For older adults, detached reappraisal had a medium positive effect (*g* = 0.62, 95% CI [0.29–0.94]), and positive reappraisal had no reliable effect (*g* = 0.10, 95% CI [−0.12–0.89]), *Q*(1) = 3.41, *p* = 0.07. We found no evidence for differences in the behavioral outcomes of expression enhancement (*g* = 0.94, 95% CI [0.48–1.39]) and expression suppression (*g* = 0.58, 95% CI [0.41–0.74]) among young adults, *Q*(1) = 2.15, *p* = 0.14. Behavioral outcomes of expression enhancement (*g* = 0.54, 95% CI [0.17–0.91]) and expression suppression (*g* = 0.52, 95% CI [0.38–0.66]) also did not differ among older adults, *Q*(1) = 0.01, *p* = 0.92.

#### Stimulus type

Next, we explored stimulus type (film vs pictures) as a potential moderator of ER success within each age group.

**Figure 3:**
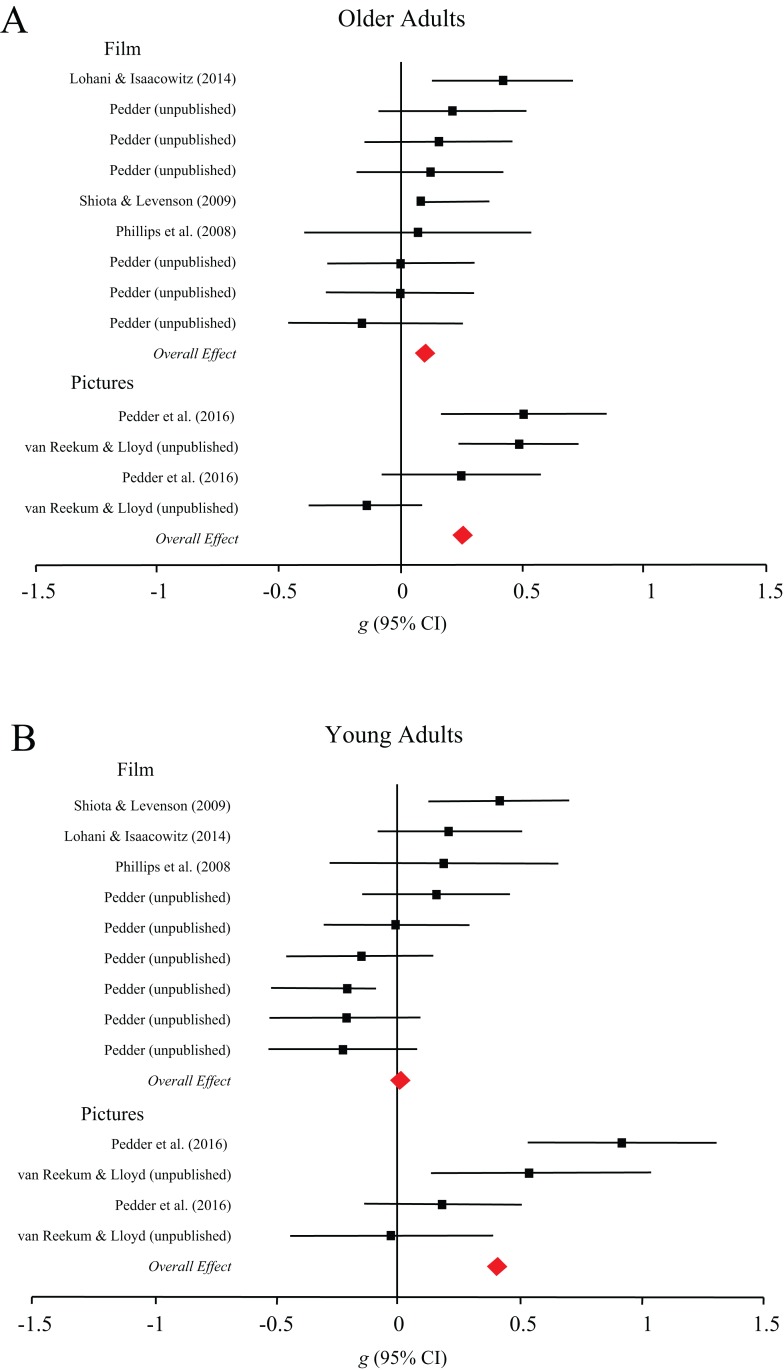
Forest plots demonstrating effect sizes for the subjective outcomes of response modulation processes for older (A) and younger (B) adults across film and picture stimuli.

**Subjective outcomes.** Stimulus type moderated the efficacy of cognitive change strategies for both young, *Q*(1) = 5.85, *p* = 0.02, and older adults, *Q*(1) = 4.34, *p* = 0.04, such that both groups successfully regulated emotional responses to pictures (*g* = 0.57, 95% CI [0.32–0.82] and *g* = 0.51, 95% CI [0.25–0.78], respectively), but not films (*g* = 0.16, 95% CI [−0.06–0.38] and *g* = 0.13, 95% CI [−0.12–0.37], respectively). As shown in Fig. 3, young adults regulated emotion using response modulation processes in response to pictures (*g* = 0.41, 95% CI [0.11–0.71]) but not films (*g* = 0.01, 95% CI [−0.17–0.20]), *Q*(1) = 4.87, *p* = 0.03, whereas older adults did not differ in their regulation in response to pictures (*g* = 0.26, 95% CI [0.04–0.48]) and film (*g* = 0.11, 95% CI [−0.05–0.26]), *Q*(1) = 1.21, *p* = 0.27.

**Figure 4:**
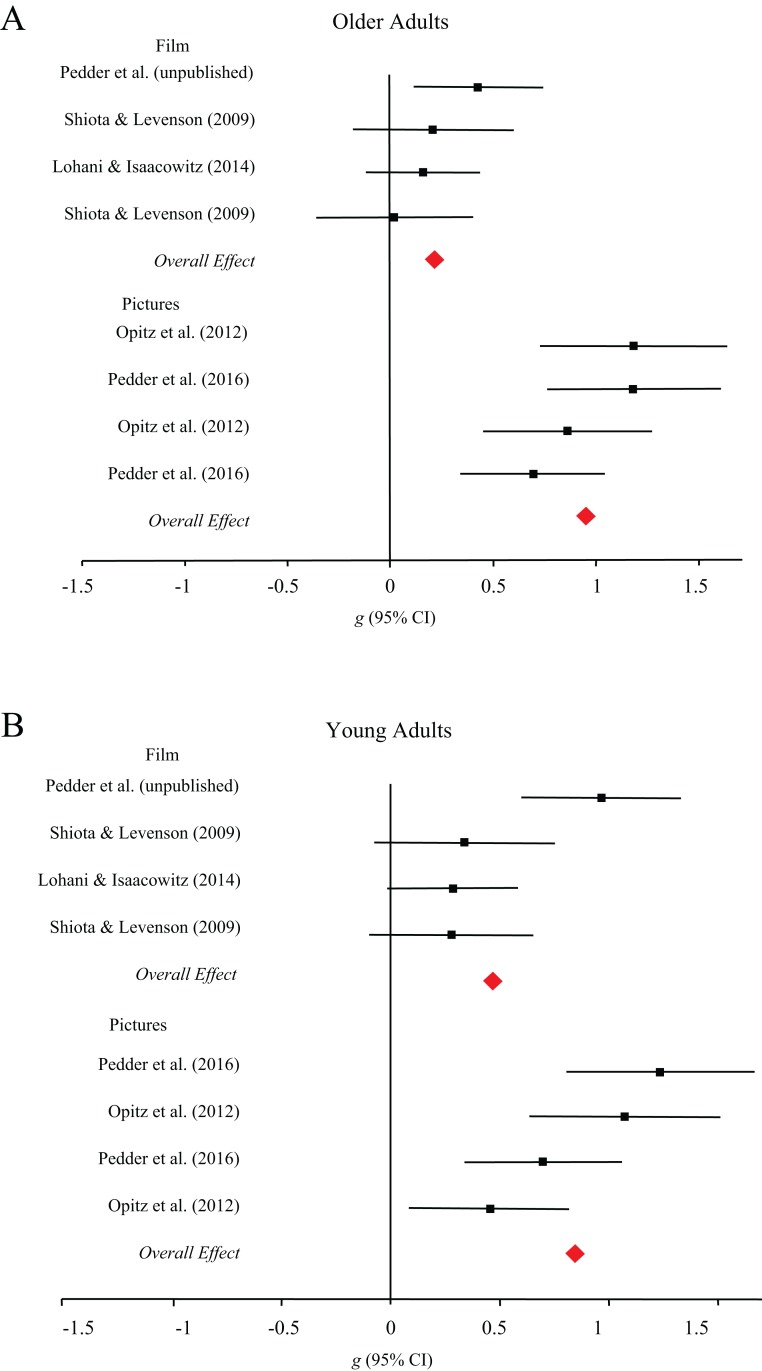
Forest plots demonstrating effect sizes for the behavioral outcomes of cognitive change processes for older (A) and younger (B) adults across film and picture stimuli.

**Behavioral outcomes.** As shown in Fig. 4, older adults were more successful at using cognitive change processes to regulate emotion elicited by pictures (*g* = 0.95, 95% CI [0.73–1.17]) compared to films (*g* = 22, 95% CI [0.03–0.40]), *Q*(1) = 25.03, *p* < 0.001. Among young adults, we found no evidence for differences in ER success across picture (*g* = 0.85, 95% CI [0.51–1.19]) and film stimuli (*g* = 0.47, 95% CI [0.13–0.80]), *Q*(1) = 2.47, *p* = 0.12. The majority of studies exploring behavioral outcomes of response modulation processes used film to elicit emotion. Music and pictures were only used in one study each, which prevented moderator analyses on the basis of stimulus type.

#### Stimulus valence

Valence of the emotional stimulus (positive compared to negative) was explored as a moderator for the subjective and behavioral outcomes of response modulation strategies. Similar analyses could not be performed for cognitive change processes, as only one study to date (i.e., Pedder et al., 2016) has investigated the efficacy of these strategies for regulating positive emotion.

**Subjective outcomes.** We found no evidence for an effect of valence on successful regulation of subjective emotion following response modulation among young (positive valence: *g* = 0.15, 95% CI [−0.18–0.48]; negative valence: *g* = 0.11, 95% CI [−0.11–0.34]), *Q*(1) = 0.03, *p* = 0.86, or older adults (positive valence: *g* = 0.24, 95% CI [0.01–0.48]; negative valence: *g* = 0.12, 95% CI [−0.04–0.27]), *Q*(1) = 0.80, *p* = 0.37.

**Behavioral outcomes.** Valence of the emotional content also did not moderate the successful regulation of behavioral outcomes following response modulation among young (positive valence: *g* = 0.59, 95% CI [0.36–0.82]; negative valence: *g* = 0.71, 95% CI [0.53–0.89]), *Q*(1) = 0.59, *p* = 0.44, or older adults (positive valence: *g* = 0.60, 95% CI [0.36–0.84]; negative valence: *g* = 0.56, 95% CI [0.38–0.75]), *Q*(1) = 0.06, *p* = 0.81.

#### Publication bias

We examined the distribution of included effect sizes in order to determine whether the results of our analyses are likely to be biased by selective publishing of significant results. Fail-safe *N* (Rosenthal, 1979) revealed that 13,827 unpublished studies with zero effect sizes would be required to overturn the conclusion that instructed ER significantly impacts emotional outcomes (pooled across subjective, behavioral and physiological measures). To investigate whether failure to publish studies with negligible results (rather than zero results) would impact the conclusions of the present meta-analysis, we used Orwin’s fail-safe *N* (Orwin, 1983) with the criterion for a trivial hedges *g* set at 0.10. This revealed that 226 unpublished studies reporting zero results would have to exist in order to reduce the overall effect size reported in the present meta-analysis to 0.10. We therefore determined that in this light and because we included unpublished studies where possible, it is unlikely that publication bias is a concern for the present meta-analysis.

## Discussion

This meta-analysis provides evidence for age-related retention of ER capacity (attentional deployment, cognitive change, and response modulation) in the subjective, behavioral, and physiological domains. A small number of age-related differences were found. Subjective outcomes indicate that within-process strategy and stimulus type moderate instructed response modulation effectiveness among young but not older adults. Specifically, younger adults successfully regulate emotion using expressive enhancement but not expressive suppression, and in response to pictures but not films. In contrast, no evidence for similar differences was found among older adults. Using instructed cognitive change strategies, older adults were better able to regulate behavioral displays of emotion in response to pictures compared to films, whereas no evidence of differences across stimuli type was found among younger adults. Valence of the emotive stimuli was not found to moderate successful response modulation for young or older adults.

## Overall Effects Across Age Groups

In line with the predictions of the process model (Gross, 1998), all ER processes had a positive effect on emotional outcomes, averaged across age group and outcome measure. Cognitive change processes had a small-to-medium effect (*g* = 0.36) and both response modulation and attentional deployment processes had small effects on emotional outcomes (*g* = 0.26 and *g* = 0.25, respectively). The present results are consistent with effect sizes reported in Webb, Miles & Sheeran’s (2012) meta-analysis. It should be noted here that Webb et al. reported that attentional deployment strategies had no effect on emotional outcomes (*d_+_* = 0.00). However, this result was driven by a positive effect size for distraction strategies (*d_+_* = 0.27), and a negative effect size for concentration strategies (*d_+_* = −0.27). The present meta-analysis included only studies investigating age-related differences in the effect of distraction strategies on emotional outcomes and the effect size is consistent with Webb et al.’s distraction effect size.

## Age-Differences in Emotion Regulation

### Attentional deployment

Older adults have been shown to use shifts in attention in order to maintain or enhance positive emotional states (Carstensen, Mikels & Mather, 2006). Indeed, attentional deployment has been proposed as a particularly useful strategy for older adults given their positive attentional preferences, and the reduced cognitive demand of attentional deployment relative to cognitive change strategies (Sheppes & Gross, 2011). However, the present meta-analysis found no evidence of age-related differences in the success of attentional deployment processes, which had positive effects on both subjective (*g* = 0.43) and behavioral outcomes (*g* = 0.37) averaged across young and older adults. This result is consistent with Webb, Miles & Sheeran’s (2012) findings regarding the effect of active positive distraction on subjective (*d*_+_ = 0.56) and behavioral (*d*_+_ = 0.54) outcomes.

It is important to note that conclusions regarding a lack of age-related differences in the success of attentional deployment strategies must be considered tentative due to the small number of studies that have explored age-related differences in instructed attentional deployment. It is also important to consider possible methodological elements that limit the ability to detect age-differences if they do exist. For example, failure to find the proposed age effects in the present meta-analysis may also be due in part to the nature of the emotive stimuli used in aging studies to date. It is not always possible to direct attention away from negative stimuli in the lab, particularly when the stimuli are comprised of both auditory and visual components (e.g., the negative films used in attentional deployment aging studies to date). Further research exploring the effect of age on attentional deployment success across a range of emotive stimuli is required in order to ascertain whether shifts in attentional preferences as we age are associated with changes in ER success.

### Cognitive change

We also found no evidence for age-related differences in the success of instructed cognitive change strategies. In line with Webb, Miles & Sheeran (2012), these strategies were found to have a positive effect on both subjective (*g* = 0.33) and behavioral outcomes (*g* = 0.61), and no reliable effect on physiological outcomes (*g* = 0.03), averaged across age group. Within-process strategy type (detached reappraisal, negative reappraisal, and positive reappraisal) was explored as a moderator of subjective and behavioral ER success among young and older adults. Based on the benefits older adults attain from positively reappraising emotional stimuli (see review by Nowlan, Wuthrich & Rapee (2015)), as well as their attentional preferences for positive emotional information (Reed, Chan & Mikels, 2014), we expected that positive reappraisal may have been more effective for older adults compared to younger adults. Instead, we found no evidence of age-related differences in the subjective outcomes of cognitive change strategies, and in direct contrast with our prediction, both age groups were more successful at regulating behavioral indicators of emotion when using detached reappraisal relative to positive reappraisal. This is consistent with Webb et al.’s finding that perspective-taking based strategies (i.e., detached reappraisal) are more effective than strategies that involve reappraising the emotional stimulus (i.e., positive reappraisal), as well as recent research demonstrating greater success using detached reappraisal compared to positive reappraisal among both young and older adults (Livingstone & Isaacowitz, 2018). It is also in line with the predictions of DIT (Labouvie-Vief, 2008) that strategies that require distancing oneself from elaborate processing of emotive stimuli will be less taxing on cognitive resources and should thus be associated with greater ER success. The current data is also consistent with one previous study finding reduced use of positive reappraisal by healthy older adults, although note that another has found no age-related difference in the frequency of positive reappraisal (see Nowlan, Wuthrich & Rapee, 2015).

Using cognitive change strategies, older adults were better at regulating behavioral expressions of emotion in response to pictures than films, whereas the type of stimulus did not influence the performance of young adults. Better regulation of emotion in response to unimodal pictorial relative to multimodal film stimuli among older adults is in line with DIT (Labouvie-Vief, 2008). It suggests that intense emotional content may result in the breakdown of systems that regulate the integration of cognition and emotion. Older adults may find it easier to regulate behavioral responses to pictures because they are not as cognitively demanding as film stimuli, and allow for capitalization on age-related strengths. This finding does not support the idea that older adults will perform better under more naturalistic conditions that are offered by multimodal relative to unimodal stimuli (Phillips et al., 2014). However, future research should assess real-life encounters to more thoroughly evaluate this claim. In addition, subjective outcomes of response modulation (described below) suggest that older and not younger adults successfully regulate emotion elicited by film. Benefits of more naturalistic stimuli may therefore depend on the regulation strategy.

### Response modulation

No evidence for overall age-related differences in the ability to regulate emotion using response modulation processes were found. Averaged across age group, response modulation strategies had a negligible positive effect on subjective outcomes (*g* = 0.12), a medium positive effect on behavioral outcomes (*g* = 0.57), and no reliable effect on physiological outcomes (*g* = 0.05). The subjective and behavioral data closely align with Webb, Miles & Sheeran’s (2012) analysis of a primarily younger cohort. However, unlike the current study, Webb et al. found that response modulation strategies had a small negative effect on physiological outcomes. This difference may be explained by the inclusion of only suppression (and not enhancement) strategies in Webb et al., whereas the present meta-analysis included strategies that regulate the emotional response via both enhancement and suppression. It is possible that suppression requires more effort than up-regulating an existing emotional response, resulting in reduced physiological effectiveness of response modulation in the previous meta-analysis.

Within-process strategy type (expressive enhancement, expressive suppression) was explored as a moderator of subjective and behavioral ER success among young and older adults. Subjective outcomes revealed that young adults effectively use expression enhancement but not suppression to regulate emotion, whereas older adults effectively use both of these strategies. Prior research has found that older adults require fewer cognitive resources to implement response-focused strategies compared to younger adults (Mather, 2012; Robinson & Demaree, 2009), are better able to tolerate a mismatch between inner experience and outer expression (Urry & Gross, 2010), may be more motivated to reduce negative expressivity compared to younger adults (Carstensen, Gross & Fung, 1997; Lawton et al., 1992), and have lower facial expressivity overall that may be easier to down-regulate as we age (Pedder et al., 2016). Combined, these factors may explain older adults’ greater success using expressive suppression to regulate subjective reactivity compared to younger adults.

Stimulus type was also explored as a moderator of response modulation success. Subjective outcomes indicate that young adults regulate emotion effectively in response to pictures but not films, while older adults can regulate both pictures and films. This contrasts with cognitive change strategies that resulted in greater success for older adults in response to pictorial relative to film stimuli. As noted, it is possible that more naturalistic multimodal film stimuli is particularly beneficial to older adults when implementing response modulation. It might also be the case that response modulation is more useful to older adults than cognitive change when dealing with more arousing stimuli.

The valence of the emotive stimuli was not found to moderate the success of response modulation strategies among young or older adults in the present meta-analysis. This is consistent with the age-related positivity effect derived from successful up-regulation of positive affect and down-regulation of negative affect in older age (Isaacowitz, 2006). More research is needed to clarify whether valence of the emotional stimuli is associated with age-related differences in the success of attentional deployment or cognitive change strategies.

## Limitations and Future Directions

Meta-analyses that include a small number of studies are sometimes criticized as premature. On the contrary, meta-analytic procedures allow us to summarize the results of as few as two or more studies with greater power than individual studies (Goh, Hall & Rosenthal, 2016). Importantly, a meta-analysis can also lend support to null effects when individual studies cannot (Goh, Hall & Rosenthal, 2016), and while the current meta-analysis consistently found null effects of age on ER success, it also consistently found that instructed regulation strategies were effectively applied by both young and older adults. It might also be argued that our power was limited by creating subcategories for moderator analyses. However, this counteracts a major criticism of meta-analysis, which is that results that differ systematically are often inappropriately combined, also known as “mixing apples and oranges.” Thus, the current data describes more than just the summary effect of age differences in response to overall emotional regulation processes. Moreover, the findings of a maintained, and sometimes enhanced, capacity for ER in older adulthood provides empirical weight to well-documented theories predicting exactly these effects. This systematic review and meta-analysis also paves the way for future directions in this critical field of research.

Age-related differences in the success of many instructed ER strategies remains under-researched. Only one unpublished study to date (D. Pedder, unpublished data) has examined age differences in the efficacy of instructed mindfulness-based ER strategies, and no study has explored age differences in instructed acceptance strategies. Future studies exploring age-related differences in the efficacy of these strategies is warranted. The present meta-analysis identified no effect of stimulus valence on young and older adults’ ER success. However, particular emotions may become more prevalent and important as we age and we may see age-related differences in the success of strategies that effectively regulate those more frequently experienced emotions. For example, the salience of anger decreases in middle and older adulthood, whereas the intensity and frequency of sadness remains stable or increases (Kunzmann, Kappes & Wrosch, 2014). Future research should explore age-related differences in the regulation of discrete emotions that may be increasingly important as we age. It will also be important to assess a broader spectrum of ages to rule out cohort effects that are inherent in extreme age group designs and to identify any differences across the entire adult lifespan, or within subgroups of older adults. Similarly, future studies would benefit from extending this work to encompass older clinical populations.

While the present meta-analysis offers insight into age differences in the success of instructed ER strategies, it does not address the success of spontaneous strategy use (where people could freely choose how to regulate emotion). In line with the predictions of SST (Charles & Carstensen, 2007), older adults may excel at spontaneous ER. Contributing to a bigger picture understanding of age-related differences in ER using procedures that strive for ecological validity is a critical avenue for research moving forward that has been recently noted in the literature (Kunzmann & Isaacowitz, 2017). To date, it is not clear whether older adults’ success using instructed ER strategies in the lab environment translates to success in the real world. The current meta-analysis provides a critical starting point for making such comparisons.

## Conclusions

Overall, the present systematic review and meta-analysis provides empirical support for theoretical accounts of maintained ER capacity in older age. The findings demonstrate a general lack of evidence for age-related differences in the success of instructed attentional deployment, cognitive change and response modulation strategies, with a few exceptions. Expression suppression was found to influence the subjective emotional response of older adults, but not younger adults. In addition, only older adults use response modulation to successfully regulate subjective emotional responses to films. Interestingly, both young and older adults’ behavior indicated effective use of detached reappraisal but not positive reappraisal. As proposed by SST (Charles & Carstensen, 2007), older adults may be more motivated to engage in ER in order to maintain emotional wellbeing compared to their younger counterparts. This increased motivation may scaffold for ER difficulties that could otherwise be predicted on the basis of reduced cognitive resources in ageing. From the perspective of SAVI (Charles, 2010), compared to younger adults, older adults’ greater experience navigating the emotional challenges of life may better prepare them for regulating emotions using a broad range of strategies. This may be particularly the case for less cognitively demanding strategies, as set out by DIT (Labouvie-Vief, 2008). Together, these theoretical accounts of social and emotional ageing provide a plausible explanation for the maintenance, and in some cases enhancement, of older adults’ responding to ER instructions in the present systematic review and meta-analysis.

## Supplemental Information

10.7717/peerj.6051/supp-1 Supplemental Information 1 Rationale and Contribution.

10.7717/peerj.6051/supp-2 Supplemental Information 2 PRISMA checklist.

## Additional Information and Declarations

### Data Availability

The following information was supplied regarding data availability: All relevant data is contained in Table 1.
