# Supplementary material for: Systematic review and meta-analysis of age-related differences in instructed emotion regulation success
Source: PeerJ. 2018 Dec 18;6:e6051. doi: 10.7717/peerj.6051 (PMC6302795; doi:10.7717/peerj.6051)
Supplement: Supplemental Information 1 [file peerj-06-6051-s001.docx]

**1. Rationale for conducting the meta-analysis.**

The global population is aging rapidly. Contrary to well-documented physical and cognitive declines in late adulthood, emotion regulation capacities have been suggested to improve in older age (Charles and Carstensen, 2007).

There is a growing body of research investigating age-related differences between young and older adults in the success of instructed emotion regulation strategies. However, no meta-analysis to date has investigated systematic age-related differences in emotion regulation success between these two age groups.

The identification of emotion regulation strategies that are particularly effective in older age has important implications for supporting healthy aging. Clarifying the state of instructed emotion regulation and aging research to date will also help to inform research directions moving forward.

**2. Contribution that the meta-analysis makes to knowledge in light of previously published related reports, including other meta-analyses and systematic reviews.**

This is the first meta-analysis to consider differences between young and older adults in the success of instructed emotion regulation strategies. This meta-analysis contributes to current understanding regarding aging and emotion regulation by supporting the notion that emotion regulation is maintained in older age, while also identifying previously understudied regulation strategies (i.e. detached reappraisal) that may be particularly beneficial for older as well as younger adults. While the number of separate studies included in this meta-analysis is relatively small (k = 11), our synthesis of published and unpublished data provides a clearer picture of aging and instructed emotion regulation than previously available.
